# Under the Radar: Back Pain and Acute Kidney Injury as Harbingers of Type A Aortic Dissection

**DOI:** 10.7759/cureus.90584

**Published:** 2025-08-20

**Authors:** Yohannes Debebe Gelan, Mark Ntow, Samuel Sule-Saa, Ajibola Adedayo

**Affiliations:** 1 Internal Medicine, Interfaith Medical Center, Brooklyn, USA; 2 Cardiology, Interfaith Medical Center, Brooklyn, USA

**Keywords:** acs, acute coronary syndrome, acute kidney injury, low back pain (lbp), systemic hypertension, type a aortic dissection

## Abstract

Aortic dissection is a catastrophic vascular emergency with a high mortality rate if not diagnosed and managed in a timely manner. The classic presentation of thoracic aortic dissection includes the sudden onset of severe chest pain radiating to the back. Still, it may also present with atypical symptoms and signs of end-organ vascular compromise.

We present a case of a 57-year-old female with a medical history of hypertension with poor medication adherence, major depressive disorder, chronic low back pain, and cocaine use disorder, who initially presented to the ED with worsening chronic low back pain, which was attributed to a musculoskeletal origin. She received analgesics, which improved the pain. Approximately 7 hours after presentation, the following morning, the patient developed acute-onset chest pain, prompting repeat evaluation, including an electrocardiogram and cardiac biomarkers, which revealed T-wave inversion in the lateral leads and elevated troponin levels. Based on these findings, the non-ST elevation myocardial infarction (NSTEMI) protocol was initiated. While the back pain and chest pain improved, her kidney function continued to rapidly deteriorate from the time of admission, prompting the team to perform a sonogram and subsequently an abdominal CT, which revealed a heterogeneous, hyperechoic right kidney with loss of corticomedullary distinction and possible aortic pathology. A CT angiogram of the chest, abdomen, and pelvis confirmed a type A aortic dissection extending from the aortic root to the iliac bifurcation. The dissection caused significant narrowing at the origin of the left common carotid artery and partial infarction of the right kidney due to involvement of the renal artery. The patient was transferred to a tertiary hospital for surgical intervention and remained hemodynamically stable; however, she opted against surgical intervention and left against medical advice despite extensive counseling. Clinicians should maintain a high index of suspicion for aortic dissection in patients with unexplained back pain, particularly when there are signs of multiorgan ischemia.

## Introduction

Aortic dissection is a rare, catastrophic vascular disorder with a reported pooled incidence of 4.8 per 100,000 per year, caused by a tear in the intimal layer of the aorta, leading to separation of the aortic luminal layers [1]. Blood dissecting between the intima and media propagates either forward or backward, compromising the blood supply to vital organs. Classically, aortic dissection presents as sudden-onset, sharp, tearing pain in the chest that radiates to the back. Many clinicians rely on the presence of characteristically severe, tearing, descending pain to suggest the diagnosis [2]; however, many patients present with atypical or subtle symptoms, leading to missed diagnoses - two-thirds of verified cases are not accurately diagnosed on initial presentation [3,4].

The Stanford classification divides aortic dissection into type A, involving the ascending aorta, and type B, confined to the descending aorta. Type A requires urgent surgery, whereas type B is usually managed medically [5]. An ascending aortic dissection is a medical emergency that can cause sudden death; without repair, mortality increases by 1-2% per hour in survivors of the acute event [6]. The importance of early recognition cannot be overstated.

Abrupt onset of pain is observed in 85.5% of patients, with chest pain in 81.6%, back pain in 45.8%, and abdominal pain in 22% of cases [7]. However, atypical presentations, including the absence of classic chest pain, contribute to diagnostic challenges. Aortic dissection is underrecognized in elderly patients, women, and those with comorbidities [8]. Sudden and severe back pain is a well-documented presenting symptom, particularly in cases involving the abdominal aorta [9,10]. Diagnosis becomes more challenging when the pain is only moderately severe, and in patients with a history of chronic back pain, new or worsening symptoms may be misattributed. In such cases, certain features, such as the unremitting nature of the pain and the presence of risk factors like hypertension or the use of sympathomimetic agents (e.g., cocaine), can serve as important diagnostic clues [11].

Additionally, manifestations from compromised blood flow to vital organs can aid in diagnosing aortic dissection. These include syncope, stroke symptoms, acute coronary syndrome (ACS), abdominal or limb pain, and signs of ischemia or acute kidney injury [12]. A missed diagnosis can be catastrophic in a patient presenting with ACS, as standard treatment involves antiplatelet and anticoagulant medications, which are absolutely contraindicated in aortic dissection because they can worsen bleeding, extend the dissection, and increase the risk of death [13]. This case illustrates an initial diagnostic challenge due to an atypical presentation of aortic dissection.

## Case presentation

A 57-year-old female with a history of hypertension, poor medication adherence, major depressive disorder, chronic back pain, cocaine use disorder, and chronic obstructive pulmonary disease (COPD) presented to the emergency department with worsening lower back pain. She reported that she had chronic back pain, but noted that this episode was more severe. She denied any recent antecedent trauma. The patient described the back pain as a muscle spasm affecting her back and upper buttocks. She stated that movements exacerbated her back pain. There was no associated abdominal pain or chest pain at the time of presentation. She denied issues with urination or defecation and did not report weakness in her legs or anesthesia in her groin.

Upon presentation, the patient was hypertensive but not tachycardic. The physical examination did not reveal asymmetric pulses or differences in blood pressure between the limbs. She had no focal neurological deficits. The back pain was initially attributed to a musculoskeletal cause. Immediate imaging was not deemed necessary because her pain improved significantly with analgesics, and she had no major red flag signs.

Approximately 7 hours later, upon awakening the next morning, the patient reported new-onset chest pain, prompting re-evaluation. She also reported that she had been experiencing nausea, dizziness, and a brief loss of consciousness one day prior to presenting to the hospital. Still, she did not seek medical attention at that time.

**Figure 1:**
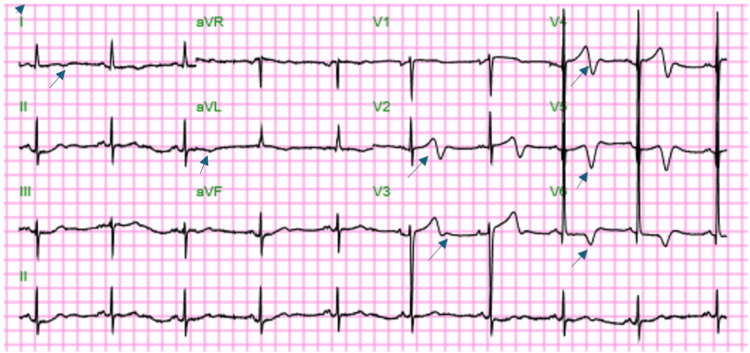
A 12-lead EKG demonstrating T-wave changes in lead I, aVL, and precordial leads.

On physical examination, she remained hemodynamically stable. She was in mild discomfort but had no focal neurological deficits, no spinal tenderness, and no fever. EKG and cardiac enzymes were obtained immediately. The ECG showed T-wave inversions in the lateral leads I, aVL, V4, V5, and V6 (Figure 1). Laboratory workup revealed elevated high-sensitivity troponin levels of 312.4 pg/mL (normal range: <5.0-11.8 pg/mL), a brain natriuretic peptide (BNP) of 217 pg/mL (normal value: <100 pg/mL), and acute kidney injury creatinine of 1.8 mg/dL (normal range: 0.6-1.1 mg/dL). Urine drug screening was positive for cocaine.

The initial evaluation raised suspicion for non-ST elevation myocardial infarction (NSTEMI), prompting admission for telemetry monitoring and further workup. The cardiology team was consulted, and she was started on aspirin, Plavix, atorvastatin, and heparin for suspected ACS, with serial troponin monitoring showing continued elevation, peaking at 5,200 ng/mL. Echocardiography revealed left ventricular hypertrophy with normal systolic function (EF 70%) and grade 1 diastolic dysfunction. Cardiac catheterization was postponed due to worsening acute kidney injury (AKI). She was continued on therapeutic anticoagulation and managed with further blood pressure optimization and analgesia.

On hospital day two, her renal function worsened (creatinine peaked at 3.2 mg/dL), prompting consultation with nephrology. Despite conservative management with IV fluids, her renal function continued to deteriorate. The patient was also noted to have a significant drop in platelet count from her admission level (195,000/µL to a nadir of 93,000/µL), raising concern for intermediate-probability heparin-induced thrombocytopenia (HIT), with a 4T score of 4. While awaiting HIT workup results, heparin was discontinued, and alternative anticoagulation with argatroban was initiated.

**Figure 2:**
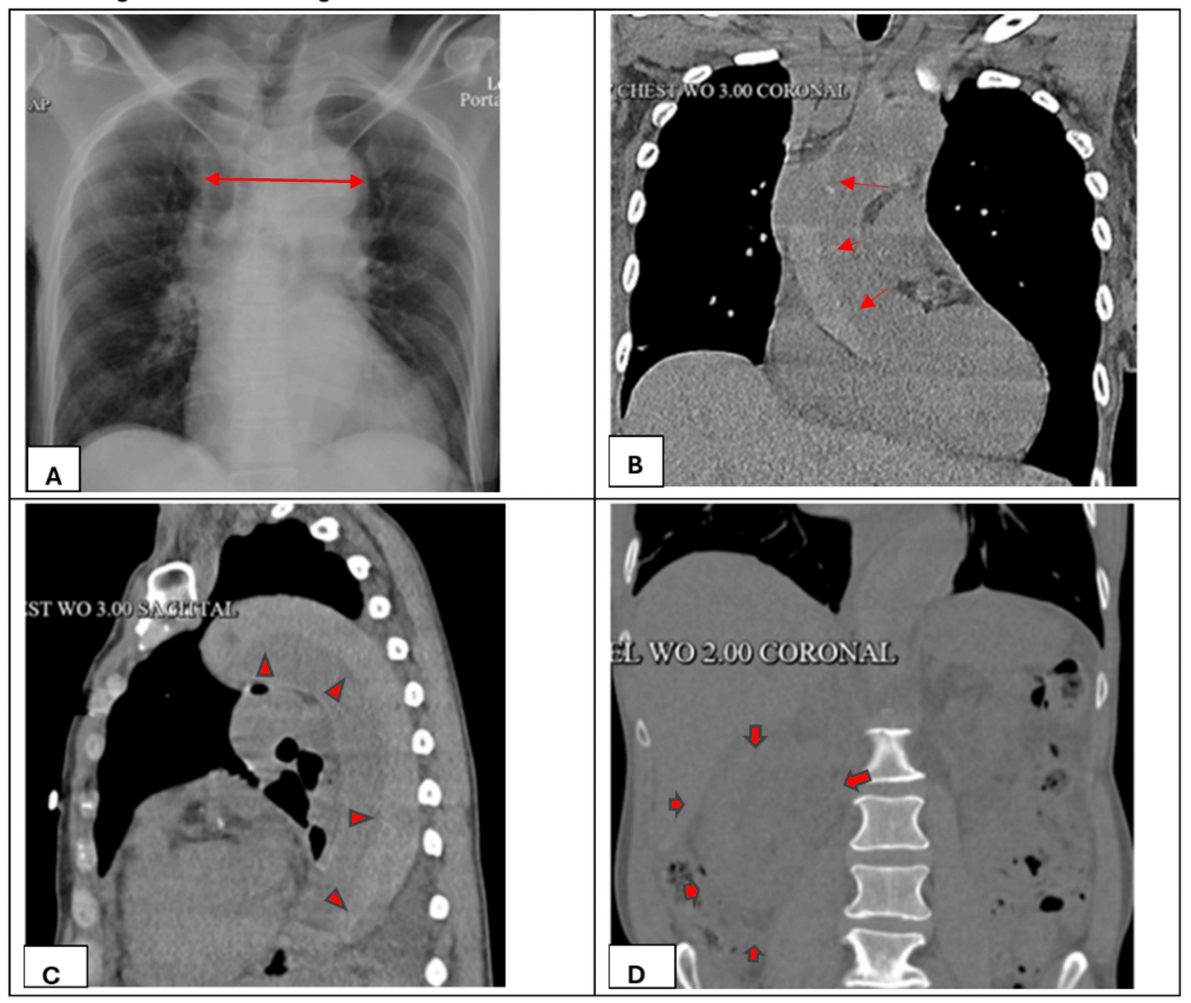
Images demonstrating extensive Stanford type A aortic dissection with signs of right kidney ischemia. (A) Chest X-ray showing mediastinal widening, initially reported as a prominent cardiomediastinal silhouette (red arrow). (B) Coronal plane CTA demonstrating a vertical intimal flap (red arrowheads) separating the true lumen from the false lumen, consistent with Stanford type A aortic dissection. (C) Sagittal plane CTA showing extensive aortic dissection extending from the aortic root to the abdominal aorta; red arrowheads indicate the dissection plane. (D) Coronal CT scan showing an enlarged right kidney with extensive perinephric fluid (red arrows). CTA: computed tomography angiography

On hospital day three, a CT scan of the abdomen and pelvis was performed after abdominal sonography revealed an abnormal heterogeneous hyperechoic right kidney with loss of corticomedullary distinction, suspicious for an infiltrative process. A chest X-ray and chest CT scan revealed aortic pathology suspicious for dissection. The X-ray findings are shown in Figure 2A. A subsequent CT angiogram of the chest, abdomen, and pelvis confirmed a type A aortic dissection extending from the aortic root through the iliac bifurcation (Figures 2B, 2C), with significant narrowing at the origin of the left common carotid artery and partial infarction of the right kidney due to involvement of the renal artery (Figure 2D).

Following the diagnosis, the patient was transferred to the intensive care unit for strict blood pressure control with a labetalol drip (goal BP: <120/80 mmHg, HR: <80 bpm) and was evaluated by the cardiothoracic surgery team. Ultimately, a decision was made to transfer the patient to a tertiary cardiac surgical center for possible intervention. The patient was transferred to a hospital with cardiothoracic surgery services and remained hemodynamically stable. She refused surgical intervention and was discharged against medical advice. Subsequent attempts to establish follow-up were unsuccessful.

## Discussion

Aortic dissection has a mortality rate of up to 50% within 48 hours. However, with prompt diagnosis and surgical intervention, the mortality rate for type A dissection can significantly drop, emphasizing the significance of early diagnosis [14]. One of the complications of aortic dissection is involvement of the coronary arteries, which is seen in about 7-20.7% of cases of acute type A aortic dissection and may create diagnostic and management dilemmas [15]. Managing acute coronary syndrome involves initiating anticoagulant therapy, which can have catastrophic consequences in a patient with an undiagnosed aortic dissection. Diagnosing NSTEMI in particular heavily relies on clinical history, cardiac biomarkers, and EKGs, while aortic dissection is confirmed through imaging [13].

The delay in diagnosis highlights the challenge of recognizing aortic dissection in patients with atypical presentations and when clinical findings point to another life-threatening emergency, such as ACS. The absence of chest pain or characteristic progressive sharp back pain at initial presentation, and subsequent overlap with ACS, led to a focus on cardiac ischemia rather than vascular pathology. Additionally, chronic back pain may have masked the acute event’s severity, and diagnostic anchoring on usual degenerative pathologies delayed further imaging that could have identified the dissection earlier.

Utilizing the Acute Aortic Dissection Risk Score ≥1 and/or a positive D-dimer can facilitate early identification of aortic dissection and guide clinicians in obtaining appropriate imaging (CT angiography or transesophageal echocardiography) [16]. It is also important to reassess initial diagnoses when new symptoms emerge or existing symptoms evolve.

The patient’s progressively worsening renal function led the team to postpone cardiac catheterization and instead pursue renal imaging. It is reported that cocaine-related acute aortic dissection is associated with a higher incidence of acute renal failure; however, despite this complication, patients with type A cocaine-related dissections tend to have significantly lower mortality rates compared to non-cocaine-related cases, likely due to the younger age group typically affected by cocaine use [11].

## Conclusions

This case underscores the importance of considering vascular emergencies, such as aortic dissection, in patients presenting with worsening or atypical back pain, especially when risk factors like hypertension and cocaine use are present. Signs of multiorgan ischemia, such as acute kidney injury and syncope, serve as important diagnostic clues. Early imaging, such as CT angiography, MRI, or transesophageal echocardiogram (TEE), should be pursued when symptoms persist or appear atypical. Careful and ongoing reassessment of diagnoses as symptoms evolve is essential to ensure timely detection and appropriate management.
